# Radiotherapy plays an important role in improving the survival outcome in patients with T1–2N1M0 breast cancer – a joint analysis of 4262 real world cases from two institutions

**DOI:** 10.1186/s12885-020-07646-y

**Published:** 2020-11-26

**Authors:** Guang-Yi Sun, Ge Wen, Yu-Jing Zhang, Yu Tang, Hao Jing, Jian-Yang Wang, Jiang-Hu Zhang, Yong Yang, Xu-Ran Zhao, Si-Ye Chen, Jing Jin, Yong-Wen Song, Yue-Ping Liu, Hui Fang, Hua Ren, Yuan Tang, Shu-Nan Qi, Ning Li, Bo Chen, Ning-Ning Lu, Shu-Lian Wang, Ye-Xiong Li

**Affiliations:** 1 State Key Laboratory of Molecular Oncology and Department of Radiation Oncology, National Cancer Center/National Clinical Research Center for Cancer/Cancer Hospital, Chinese Academy of Medical Sciences and Peking Union Medical College, Beijing, 100021 China; 2 Department of Radiation Oncology, Sun Yat-sen University Cancer Center, State Key Laboratory of Oncology in South China, Collaborative Innovation Center of Cancer Medicine, Guangzhou, 510060 China; 3 Department of Radiation Oncology, The Third Affiliated Hospital of Guangzhou Medical University, Guangzhou, 510150 China

**Keywords:** Breast neoplasm, Breast conserving surgery, Mastectomy, One to three positive nodes, Radiotherapy

## Abstract

**Background:**

To compare the survival outcomes between breast-conserving surgery (BCS) and modified radical mastectomy (MRM), and to investigate the role of radiotherapy (RT) in patients with pT1–2N1M0 breast cancer.

**Methods:**

A total of 4262 women with T1–2N1M0 breast cancer treated at two institutions were retrospectively reviewed. A total of 3858 patients underwent MRM, and 832 (21.6%) of them received postoperative RT (MRM + RT). A total of 404 patients received BCS plus postoperative RT (BCS + RT). All patients received axillary lymph node dissection, while 3.8% of them had upfront sentinel node biopsy. The association of survival outcomes with different surgical modalities (BCS vs. MRM) and the role of RT were evaluated using multivariable proportional hazards regression and confirmed by the propensity score-matching (PSM) method.

**Results:**

At a median follow-up of 71 months (range of 6–230 months), the 5-year overall survival (OS) rates of the BCS and MRM groups were 96.5 and 92.7%, respectively (*P* = .001), and the corresponding 5-year disease-free-survival (DFS) and locoregional recurrence (LRR) rates were 92.9 and 84.0%, and 2.0 and 7.0% (*P* = .001), respectively (*P* < .001). Multivariate analysis revealed that RT was an independent prognostic factor for improved OS (*P* = .001) and DFS (*P* = .009), and decreased LRR (*P* < .001). However, surgery procedure was not independently associated with either OS (*P* = .495), DFS (*P* = .204), or LRR (*P* = .996), which was confirmed by PSM analysis.

**Conclusion:**

Postoperative radiotherapy rather than the surgery procedures was associated with superior survival outcomes in patients with T1–2N1M0 breast cancer.

## Background

Early randomized trials have demonstrated that breast-conserving surgery (BCS) combined with postoperative radiotherapy (RT) can achieve equivalent overall survival compared with MRM for early-stage breast cancer patients [1–5]. Thus, the concept of “less is more” has been widely accepted by surgeons. Currently, some retrospective studies based on the real world population have found that breast-conserving therapy can result in more survival benefits to early-stage breast cancer patients than MRM (Table 1), [6–10] but the reason is unclear. The use of BCS has been relatively low in China [12], and the role of postmastectomy radiotherapy (PMRT) is controversial in T1–2N1M0 breast cancer [13, 14]. Therefore, we conducted a retrospective analysis to compare the survival outcomes between BCS + RT and MRM patients, and investigated the role of RT in patients with T1–2N1M0 breast cancer in a real world setting. Our hypothesis was that patients treated with BCS + RT had superior survival outcomes and that radiotherapy, rather than surgery procedures, contributed to the improved survival.

**Table 1:** Retrospective studies based on the real world population

Studies	Study period	Total number of patients	Stage	Group	Breast cancer death HR (95%CI)
Hwang, 2013 [6]	1990–2004	112,154	I-II	MRM ± RT	1.00
				BCS + RT	0.84 (0.78–0.91)
Agarwal, 2014 [7]	1998–2008	132,149	I-II (tumor size ≤4 cm)	BCS + RT	1.00
				MRM without RT	1.31 (1.25–1.39)
				MRM + RT	1.47 (1.34–1.61)
Hartmann-Johnsen, 2015 [8]	1998–2008	13,015	I-II (T1–2N0-1M0)	BCS + RT	1.00
				MRM ± RT	1.64 (1.43–1.88)
Hofvind, 2015 [9]	2005–2011	9.547	I-III	BCS + RT	1.00
				MRM ± RT	1.7 (1.3–2.4)
van Maaren, 2016 [10]	2000–2004	37,207	I-II (T1–2N0-1M0)	MRM ± RT	1.00
				BCS + RT	0.81 (0.78–0.85)
Christiansen, 2018 [11]	1995–2012	58,331	I-III	BCS ± RT	1.00
				MRM ± RT	1.20 (1.15–1.25)

*Abbreviations: MRM* Modified radical mastectomy, *BCS* Breast-conserving surgery, *RT* Radiotherapy

## Methods

The study protocol was approved by the Institutional Review Board of Cancer Hospital, Chinese Academy of Medical Sciences and Peking Union Medical College (approval number 15–057/984). No informed consent was sought. A total of 4262 women with pT1–2N1M0 breast cancer treated at two institutions in China between January 1999 and December 2014 were retrospectively reviewed. All patients received lumpectomy or mastectomy and axillary lymph node dissection without neoadjuvant chemotherapy. Primary tumors were 5 cm or less with one to three positive axillary lymph nodes. All patients that underwent BCS received radiotherapy. PMRT was given to patients who had more high-risk factors, such as younger age, T2, 2–3 positive nodes, less than 10 nodes dissected, lymphovascular invasion (LVI), grade 3, and estrogen receptor (ER) negative. Clinicopathological data were recorded, including age, date of surgery, tumor morphology, LVI, histological grade, tumor size, nodal status, ER status, progesterone receptor (PR) status, human epidermal growth factor receptor 2 (HER2) status, and information from adjuvant treatments.

Locoregional recurrence (LRR) was defined as a recurrence in the breast/chest wall or in ipsilateral axillary, internal mammary, or supra−/infraclavicular nodes. Overall survival (OS) was defined as the time from the date of the definitive surgery until death from any cause. Disease-free survival (DFS) was defined as the time from the date of the definitive surgery to death or first breast cancer recurrence. The general characteristics of the subjects were expressed as frequencies and percentages and compared using the Fisher exact or χ^2^ test. Survival rates were calculated by using the Kaplan-Meier method and compared by log-rank test. The association of survival outcomes with potential prognostic factors was tested by univariate Cox regression analysis and further evaluated using multivariable proportional hazards regression. To minimize differences in distribution of covariates between groups, a propensity score matching (PSM) was used which was computed taking into consideration all the possible relevant factors (Table 2) in the analysis. The matching approach was 1:1 nearest neighbor with a caliber of 10%. Statistical analyses were performed using the SPSS Package for Windows, version 23.0 (SPSS Inc., Chicago, IL, USA). A *P*-value of ≤ .05 was considered statistically significant.

**Table 2:** Baseline characteristics of the entire patient cohort

	No. (%)			***P***
	the entire cohort (***n*** = 4262)	MRM ± RT (***n*** = 3858)	BCS + RT (***n*** = 404)	
Year				< .001
1999–2008	1976 (46.4)	1871 (48.5)	105 (26.0)	
2009–2014	2286 (53.6)	1987 (51.5)	299 (74.0)	
Age (years)				< .001
≤ 40	802 (18.8)	678 (17.6)	124 (30.7)	
> 40	3460 (81.2)	3180 (82.4)	280 (69.3)	
Tumor location				.755
Inner quadrant	909 (21.3)	819 (21.2)	90 (22.3)	
Other quadrants	3292 (77.2)	2985 (77.4)	307 (76.0)	
Unknown	61 (1.4)	54 (1.4)	7 (1·.7)	
T stage				< .001
T1	2009 (47.1)	1725 (44.7)	284 (70.3)	
T2	2253 (52.9)	2133 (55.3)	120 (29.7)	
SLNB				< .001
No	4099 (96.2)	3782 (98.0)	317 (78.5)	
Yes	163 (3.8)	76 (2.0)	87 (21.5)	
No. of ALND				.834
≤ 19	2721 (63.8)	2465 (63.9)	256 (63.4)	
> 19	1541 (36.2)	1393 (36.1)	148 (36.6)	
No. of positive nodes				.016
1	2198 (51.6)	1963 (50.9)	235 (58.2)	
2	1264 (29.7)	1156 (30.0)	108 (26.7)	
3	800 (18.8)	739 (19.2)	61 (15.1)	
Lymphovascular invasion				< .001
No	3457 (81.1)	3109 (80.6)	348 (86.1)	
Yes	522 (12.2)	470 (12.2)	52 (12.9)	
unknown	283 (6.6)	279 (7.2)	4 (1.0)	
Histological grade				< .001
I	133 (3.1)	104 (2.7)	29 (7.2)	
II	2290 (53.7)	2035 (52.7)	255 (63.1)	
III	1030 (24.2)	937 (24.3)	93 (23.0)	
unknown	809 (19.0)	782 (20.3)	27 (6.7)	
Chemotherapy				< .001
No	239 (5.6)	236 (6.1)	3 (0.7)	
Yes	3995 (93.7)	3594 (93.2)	401 (99.3)	
unknown	28 (0.7)	28 (0.7)	0 (0)	
Chemotherapeutic drug				< .001
Taxane-based	2684 (63.0)	2344 (60.8)	340 (84.2)	
Others	1105 (25.9)	1057 (27.4)	48 (11.9)	
Unknown	473 (11.1)	457 (11.8)	16 (4.0)	
Hormone receptor & Hormonal therapy				< .001
negative & no	913 (21.4)	862 (22.3)	51 (12.6)	
positive & yes	2862 (67.2)	2537 (65.8)	325 (80.4)	
positive & no	312 (7.3)	295 (7.6)	17 (4.2)	
Unknown	175 (4.1)	164 (4.3)	11 (2.7)	
HER2 & Target therapy				< .001
negative & no	2846 (66.8)	2545 (66.0)	301 (74.5)	
positive & yes	233 (5.5)	198 (5.1)	35 (8.7)	
positive & no	619 (14.5)	582 (15.1)	37 (9.2)	
unknown	564 (13.2)	533 (13.8)	31 (7.7)	

*Abbreviations: MRM* Modified radical mastectomy, *BCS* Breast-conserving surgery, *RT* Radiotherapy, *SLNB* Sentinel lymph node biopsy, *ALND* Axillary lymph node dissection, *HER2* Human epidermal growth factor receptor 2

## Results

### Patient characteristics

Table 2 shows the demographic, tumor, and treatment characteristics of the entire patient cohort. The median age was 48 years old (range of 23–84 years old). All patients received axillary lymph node dissection, while 3.8% of them had upfront sentinel node biopsy. The median number of positive nodes was one (range of 1–3); the median number of dissected nodes was 17 (range of 1–59). A total of 3858 (90.5%) patients underwent MRM and 404 (9.5%) patients underwent BCS. The BCS group had more patients treated between 2009 and 2014 compared with the MRM group. A higher number of patients in the BCS group were ≤ 50 years old, showed potentially favorable characteristics, such as T1 disease, had one positive node, an absence of LVI, grade 1–2 tumors, and positive hormonal receptors compared with MRM group. There were more patients who received chemotherapy, hormone therapy, and anti-HER2 targeted therapy in the BCS group than the MRM group.

Among the 3858 patients who underwent MRM, 832 (21.6%) received postoperative RT (MRM + RT). The chest wall was irradiated in 832/832 (100%) patients, supra−/infraclavicular nodal region was irradiated in 821/832 (98.7%) patients, axilla was irradiated in 49/832 (5.9%) patients, and internal mammary chain was irradiated in 79/832 (9.5%) patients. The median total dose was 50 Gy (range of 46.8–70 Gy) using conventional fractionation in 789/832 (94.8%) patients and 43.5 Gy (range of 40–43.5 Gy) in 15 fractions in 43/832 (5.2%) patients. A total of 516/832 (62.0%) patients had information on RT techniques, of which 501 (97.1%) received two-dimensional radiotherapy, 5 (1.0%) received three-dimensional conformal radiotherapy, and 10 (1.9%) received intensity-modulated radiotherapy.

All 404 patients who underwent BCS received postoperative RT. The whole breast was irradiated in 404/404 (100%) patients, tumor bed boost was delivered in 365/404 (90.3%) patients, supra−/infraclavicular nodal region was irradiated in 107/404 (26.5%) patients, axilla was irradiated in 3/404 (0.7%) patients, and internal mammary chain was irradiated in 3/404 (0.7%). The median dose to the whole breast ± nodal regions was 50 Gy (range of 48–50 Gy) using conventional fractionation in 360/404 (90.1%) patients and 43.5 Gy in 15 fractions in 44/404 (10.9%) patients. The median dose to the tumor bed was 10 Gy (range, 10–20 Gy) using conventional fractionation in 321/365 (87.9%) patients and 8.7 Gy in three fractions in 44/365 (12.1%) patients. A total of 236/404 (58.4%) patients had information on RT techniques, of which 170 (72.0%) received three-dimensional conformal radiotherapy or intensity-modulated radiotherapy, and 66 (28.0%) received two-dimensional radiotherapy.

Among the entire cohort, 3995 (93.8%) patients received adjuvant chemotherapy, with a median of six cycles (range of 1–20). A total of 2482 (62.1%) patients received anthracycline and taxane-based regimens, 865 (21.7%) patients received anthracycline-based regimens, 135 (3.4%) patients received taxane-based regimens, 240 (6.0%) patients received other regimens, and 208 (5.2%) patients received an unknown regimen. A total of 3296/4262 (77.3%) patients had ER and / or PR positive disease, of which 2862/3296 (86.8%) received hormonal therapy. The median duration of hormonal therapy was 45 months (range of 1–180). A total of 859/4262 (20.2%) patients had HER2 positive disease, only 233/859 (27.1%) received anti-HER2 targeted therapy.

### Outcome and prognosis

**Fig. 1:**
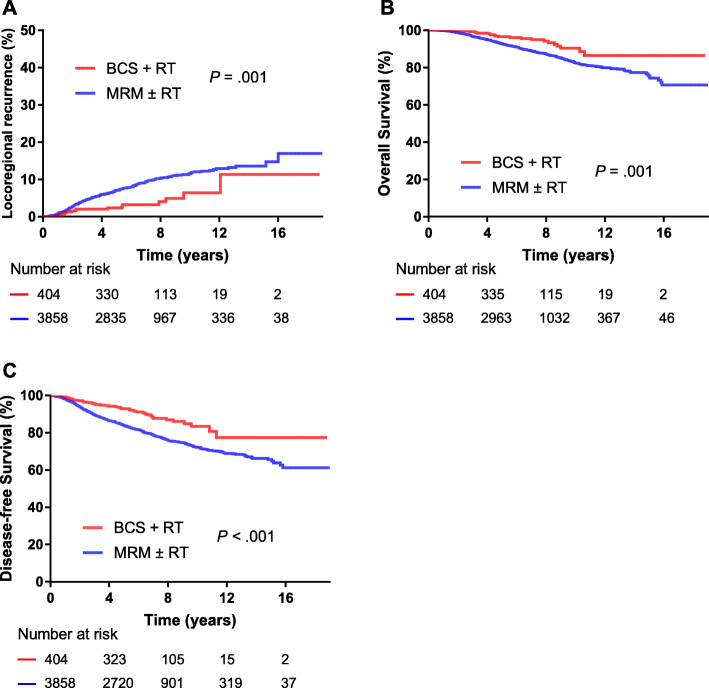
Kaplan–Meier plots showing locoregional recurrence, overall survival and disease-free survival between the BCS + RT and MRM patient groups. MRM = modified radical mastectomy; BCS = breast-conserving surgery; RT = radiotherapy.

At the median 71-month (range of 6–230 months) follow-up, 332 (7.8%) patients had locoregional recurrences, whereas 601 (14.1%) had distant metastases, and 442 (10.4%) patients had died. Among the 442 patients who died, 483 (86.7%) died from breast cancer, 4 (0.9%) died from treatment complications, 47 (10.6%) died from other causes, and 8 (1.8%) died from unknown reasons. There was no significant difference in the proportion of patients who died from breast cancer between the MRM and the BCS + RT group (86.9% vs.95.5%, *P* = .483). The 5-year LRR, OS, DFS rates were 2.0 and 7.0% (*P* = .001), 96.5 and 92.7% (*P* = .001), and 92.9 and 84.0% (*P* < .001) for the BCS + RT group and the MRM group, respectively (Fig. 1).

The univariate and multivariate analyses of prognostic factors for LRR, OS and DFS are shown in Table 3. RT was an independent prognostic factor for decreased LRR (*P* < .001) and improved OS (*P* = .001) and DFS (*P* = .009). Surgery procedure (BCS vs. MRM) was not associated with either LRR (*P* = .996), OS (*P* = .495) or DFS (*P* = .204).

**Table 3:** Univariate and multivariate analysis of risk factors for locoregional recurrence (LRR), overall survival (OS) and disease-free survival (DFS) in entire cohort

Variables	Univariate analysis		Multivariate analysis		Univariate analysis		Multivariate analysis		Univariate analysis		Multivariate analysis	
	5-year LRR % (events)	***P***	HR (95%CI)	***P***	5-year OS % (events)	***P***	HR (95%CI)	***P***	5-year DFS % (events)	***P***	HR (95%CI)	***P***
Treatment center												
Cohort 1	6.1 (152)		1.00		93.9 (148)		1.00		85.5 (367)		1.00	
Cohort 2	7.5 (97)	.229	0.94 (0.73–1.23)	.675	91.6 (105)	.012	1.08 (0.86–1.37)	.491	83.5 (216)	.672	0.87 (0.73–1.04)	.122
Year												
1999–2008	7.6 (142)		1.00		91.5 (158)		1.00		82.8 (328)		1.00	
2009–2014	5.6 (107)	.033	1.00 (0.76–1.33)	.980	94.9 (95)	< .001	0.79 (0.61–1.03)	.079	86.7 (255)	< .001	0.88 (0.74–1.06)	.187
Age (years)												
≤ 40	9.6 (65)		1.00		92.6 (52)		1.00		81.0 (134)		1.00	
> 40	5.9 (184)	.002	0.61 (0.48–1.80)	< .001	93.2 (201)	.184	0.77 (0.61–0.97)	.026	85.8 (449)	.013	0.74 (0.62–0.87)	< .001
T stage												
T1	3.9 (70)		1.00		94.9 (87)		1.00		89.6 (188)		1.00	
T2	9.1 (179)	< .001	1.87 (1.47–2.38)	< .001	91.4 (166)	< .001	1.58 (1.28–1.93)	< .001	80.6 (396)	< .001	1.58 (1.36–1.83)	< .001
SLNB												
No	7.6 (240)		1.00		92.9 (250)		1.00		84.7 (568)		1.00	
Yes	6.2 (9)	.635	2.24 (1.23–4.09)	.008	98.1 (3)	.046	0.83 (0.36–1.92)	.670	90.1 (15)	.103	1.11 (0.69–1.80)	.659
No. of ALND												
≤ 19	7.2 (73)		1.00		92.3 (183)		1.00		83.4 (411)		1.00	
> 19	5.5 (76)	.200	0.85 (0.66–1.08)	.186	94.6 (70)	.012	0.78 (0.63–0.97)	.029	87.7 (171)	.008	0.82 (0.70–0.96)	.013
No. of positive nodes												
1	5.8 (112)		1.00		94.0 (115)		1.00		85.8 (281)		1.00	
2	7.1 (80)		1.53 (1.19–1.97)	.001	92.9 (77)		1.21 (0.97–1.51)	.084	84.5 (179)		1.18 (1.01–1.39)	.041
3	8.0 (57)	.002	1.77 (1.33–2.36)	< .001	90.8 (61)	.049	1.43 (1.12–1.84)	.004	82.9 (123)	.006	1.32 (1.09–1.58)	.004
Lymphovascular invasion												
No	6.1 (189)		1.00		93.5 (195)		1.00		85.8 (447)		1.00	
Yes	8,7 (37)		1.27 (0.92–1.75)	.146	91.5 (32)		1.03 (0.75–1.42)	.851	80.7 (82)		1.16 (0.93–1.44)	.196
Unknown	8.5 (23)	.053	0.76 (0.48–1.21)	.254	90.4 (26)	.100	0.92 (0.64–1.33)	.655	80.3 (54)	.056	0.92 (0.69–1.24)	.590
Histological grade												
I- II	4.8 (103)		1.00		94.6 (110)		1.00		87.8 (266)		1.00	
III	10.3 (83)		1.41 (1.08–1.84)	.011	91.4 (76)		1.35 (1.06–1.71)	.014	81.6 (172)		1.20 (1.01–1.43)	.042
Unknown	8.6 (63)	< .001	1.21 (0.87–1.69)	.259	90.7 (67)	< .001	1.03 (0.77–1.37)	.851	80.5 (145)	< .001	1.10 (0.89–1.35)	.389
Surgery type												
MRM	7.0 (240)		1.00		92.7 (241)		1.00		84.0 (557)		1.00	
BCS	2.3 (9)	.001	1.00 (0.54–1.86)	.996	96.5 (12)	.001	1.19 (0.72–1.99)	.495	92.9 (27)	< .001	0.79 (0.55–1.14)	.204
Radiotherapy												
No	7.6 (209)		1.00		92.5 (202)		1.00		84.1 (445)		1.00	
Yes	3.8 (40)	< .001	0.42 (0.29–0.59)	< .001	94.7 (51)	< .001	0.62 (0.46–0.83)	.001	86.8 (138)	.001	0.77 (0.64–0.94)	.009
Chemotherapy												
No	4.7 (10)		1.00		89.1 (23)		1.00		83.9 (35)		1.00	
Yes	6.7 (239)		0.96 (0.44–2.09)	.922	93.3 (229)		0.40 (0.22–0.71)	.002	84.9 (546)		0.52 (0.33–0.80)	.003
Unknown	0 (0)	.546	0.58 (0.07–4.55)	.606	94.4 (1)	< .001	0.23 (0.03–1.69)	.149	88.9 (2)	.003	0.28 (0.07–1.17)	.081
Chemotherapeutic drug												
Taxane-based	6.1 (142)		1.00		94.5 (123)		1.00		86.4 (324)		1.00	
Others	8.6 (88)		1.42 (1.08–1.87)	.011	90.6 (95)		1.22 (0.96–1.56)	.103	80.7 (201)		1.20 (1.01–1.44)	.041
Unknown	4.5 (19)	.005	0.87 (0.48–1.57)	.614	91.4 (35)	< .001	1.11 (0.66–1.88)	.692	86.3 (58)	< .001	0.89 (0.61–1.30)	.551
Hormone receptor & Hormonal therapy												
negative & no	13.0 (106)		1.00		85.9 (108)		1.00		73.2 (223)		1.00	
positive & yes	4.6 (118)		0.52 (0.40–0.66)	< .001	95.6 (108)		0.44 (0.35–0.55)	< .001	89.0 (286)		0.55 (0.47–0.65)	< .001
positive & no	6.5 (18)		0.49 (0.30–0.78)	.003	90.5 (26)		0.78 (0.56–1.09)	.153	81.7 (53)		0.67 (0.50–0.89)	.005
Unknown	6.1 ()	< .001	0.79 (0.43–1.47)	.465	90.9 (11)	< .001	0.59 (0.34–1.02)	.060	82.4 (21)	< .001	0.74 (0.50–1.11)	.144
HER2 & Target therapy												
negative & no	5.7 (145)		1.00		94.1 (147)		1.00		86.5 (352)		1.00	
positive & yes	4.9 (10)		0.84 (0.46–1.53)	.578	98.6 (3)		0.44 (0.20–0.93)	.032	90.2 (19)		0.63 (0.41–0.98)	.038
positive & no	12.0 (63)		1.55 (1.18–2.04)	.002	87.7 (62)		1.35 (1.05–1.73)	.017	75.7 (133)		1.33 (1.10–1.60)	.003
Unknown	6.1 (31)	< .001	1.07 (0.77–1.50)	.677	91.4 (41)	< .001	1.13 (0.86–1.49)	.374	84.3 (79)	< .001	1.03 (0.83–1.27)	.788

*Abbreviations: HR* Hazard ratio, *CI* Confidence interval, *SLNB* Sentinel lymph node biopsy, *ALND* Axillary lymph node dissection, *HER2* Human epidermal growth factor receptor 2

### Comparison of survival outcomes between subgroups with propensity score analysis

**Fig. 2:**
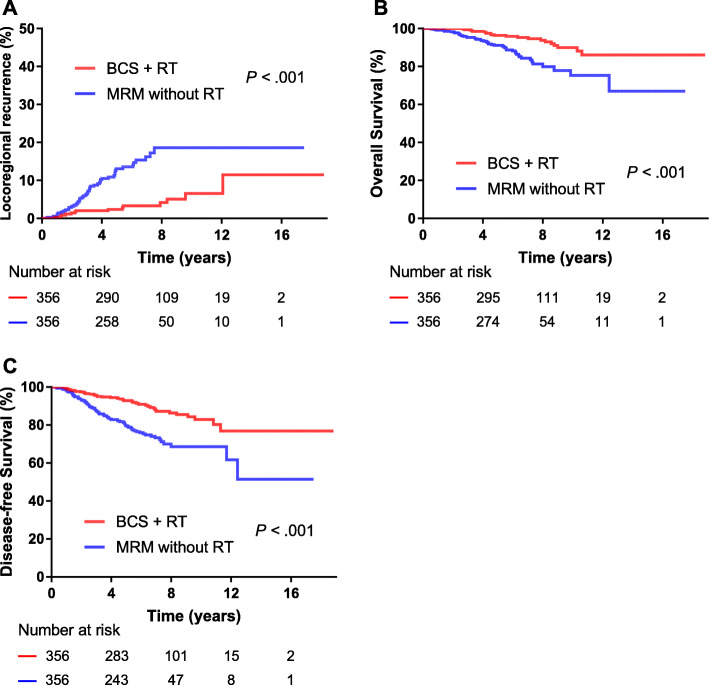
Kaplan–Meier plots showing locoregional recurrence, overall survival and disease-free survival between the BCS + RT group and the MRM without RT group. MRM = modified radical mastectomy; BCS = breast-conserving surgery; RT = radiotherapy.

**Fig. 3:**
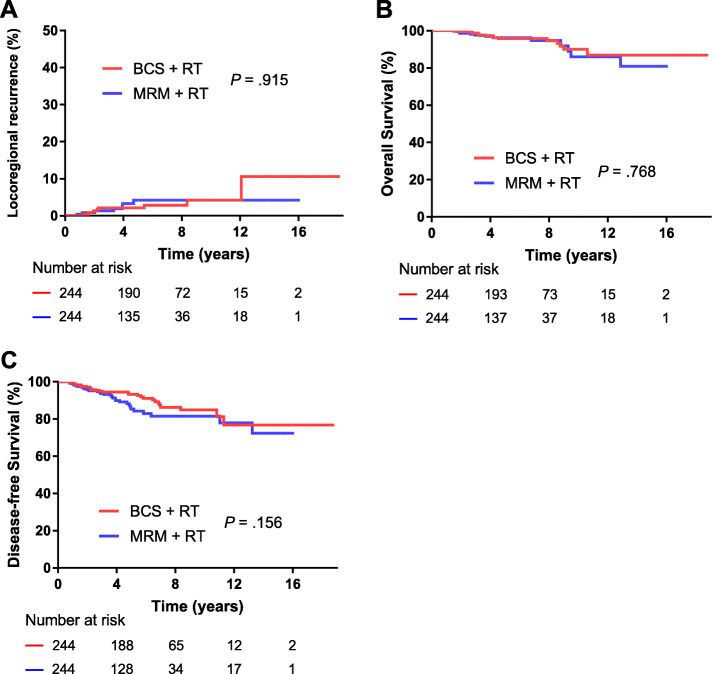
Kaplan–Meier plots showing locoregional recurrence, overall survival and disease-free survival between the MRM with RT and BCS with RT groups. MRM = modified radical mastectomy; BCS = breast-conserving surgery; RT = radiotherapy.

The demographics, tumor, and treatment characteristics are summarized and matched by propensity score analysis between BCS + RT and MRM without RT groups, and between BCS + RT and MRM + RT groups (Tables S1, S2). The characteristics were well balanced between the groups post-match. After match, BCS + RT group showed a significantly lower 5-year LRR rate (2.4% vs. 13.1%, *P* < .001), and higher 5-year OS rate (96.3% vs. 91.1%; *P* < .001) and DFS rate compared with MRM without RT group (92.9% vs. 79.7%, respectively; *P* < .001) (Fig. 2). However, there was no significant difference in 5-year LRR rate (2.1% vs. 4.2%, *P* = .915), OS rate (95.8% vs. 96.2%, *P* = .768) or DFS rate between BCS + RT and MRM + RT groups (93.3% vs. 85.3%, respectively; *P* = .156) (Fig. 3).

## Discussion

The present study compared the efficacy of BCS + RT with MRM in T1–2N1M0 breast cancer patients. The 5-year OS and DFS rates in the BCS + RT group were significantly higher than the MRM group. BCS + RT group had more favorable characteristics compared with the MRM group. Multivariate analysis revealed that the breast surgery procedure was not independently associated with patient survival. Further PSM analysis showed that the BCS + RT group had comparable survival outcomes with the MRM + RT group, and patients without RT exhibited worse survival rates than those that received RT regardless of surgery procedures.

It has been demonstrated in early randomized controlled studies [15] that BCS + RT is at least equivalent, or in recent population-based retrospective studies [6, 7, 11, 16, 17], that BCS + RT is even superior to mastectomy. Although the findings of randomized controlled trials are often considered high-level clinical evidences, their specialized research environment may differ from the environment in which large populations are located [18]. The randomized controlled studies based on a specific patient population and a specific research environment may not truly reflect the actual medical environment, the process of diagnosis and treatment, and the health status of patients under real conditions, thus leading to the failure to achieve the same results in the real world.

The present study explored why BCS + RT is superior to mastectomy, focusing on patients with T1–2N1M0 breast cancers. Multivariate analysis and PSM were performed to minimize the effects of confounding due to differences in the distribution of risk factors between treatment groups. Results showed that the survival benefit of BCS over MRM appears to be related to the combination of BCS and adjuvant RT rather than the surgical procedure. Similarly, previous reports have shown that BCS + RT resulted in better overall survival than mastectomy without RT in patients with stage I-II [6] or stage I-III breast cancer [16, 19] after accounting for factors related to treatment selection. Kim et al. found that in patients with T1–2N1 triple-negative breast cancer, BCS + RT provided significantly higher OS than MRM without RT. [20] In contrast, a Dutch study showed that, after adjusting for confounders, BCS + RT improved 10-year breast cancer-specific survival compared with mastectomy without RT, however the difference was only observed in a subset of patients with T1N0 stage disease, not in patients with T1N1, T2N0, or T2N1 stages of disease [10]. One explanation for this inconsistent finding compared with our study might be that 52% of patients in the T1–2N1 subgroup had not received chemotherapy (compared with 6% of patients in our study), as the survival benefit of RT is dependent on well-controlled distant disease with adequate systemic therapy. Recently, using a larger number of samples, a Dutch study showed superior long-term breast cancer-specific survival with BCS + RT than mastectomy ± RT in patients with T1N1 and T2N1 diseases, but the use of postmastectomy radiotherapy was not analyzed separately [21].

The present study indicated that radiotherapy played an important role in improving the survival outcomes for patients with T1–2N1 breast cancer, and clinicians should be cautious to omit radiotherapy after mastectomy in this group, although increasing data showed low-risk of locoregional recurrences in T1–2N1 breast cancer treated with modern systemic therapy [22–24]. We found that the BCS + RT group had comparable survival outcomes with the MRM + RT group, but the radiation volume in the two groups was not the same. Less proportion of patients in the BCS + RT group received regional nodal irradiation (RNI) than in the MRM + RT group. This might be due to the fact that there were more patients with favorable prognostic factors in the BCS + RT group than in the MRM + RT group, reflecting the controversy and the selective use of RNI in N1 patients within our practice. In addition, RNI was mainly delivered to the supra−/infraclavicular region, and less than 10% of patients received internal mammary nodal irradiation. If the patients in this study had received comprehensive RNI including internal mammary nodes, the more improved outcomes of RT group could have been observed, because DFS improvement resulting from supraclavicular plus internal mammary nodal irradiation was found in two recent randomized studies [25, 26].

To our knowledge, the present study is one of the few studies that has investigated the role of both surgical procedure and radiotherapy in patients with T1–2N1 stage breast cancer. An important strength of our study was the inclusion of data from two large cancer centers to thoroughly investigate the role of radiotherapy by comparing the survival outcomes between three groups, including BCT + RT, MRM with RT, and MRM without RT. Furthermore, our study accounts for as many confounding factors as possible. Although, adjusting for prognostic risk factors did not completely reduce the selection bias. Christiansen et al. reported that patients with more comorbidity were preferably treated by mastectomy, which reduced the survival in the mastectomy group [11]. In the present study, the death was mostly due to breast cancer and the OS rates likely reflect breast cancer-specific survival. In addition, disease-free survival was analyzed in our study to estimate treatment effects more reliably than overall survival by eliminating the influence of other factors leading to non-breast cancer deaths, such as comorbidities. The consistent conclusions of treatment effects on DFS and OS in the present study result in more robust findings.

Some limitations of our study should be noted. First, the proportion of BCS vs MRM in this cohort was 10% vs. 90%, which is opposite to what we see in the real world (BCS vs. MRM is usually 70% vs. 30%). However, the study using the National Cancer Data Base demonstrated increasing mastectomy rates in patients eligible for BCS with coincident increases in breast reconstruction and bilateral mastectomy [27]. Thus, the findings might be clinically relevant not only in China but also in the rest of world. Second, after developments in diagnostic and therapeutic strategies, treatment guidelines for patients with breast cancer have changed and our study population may not reflect the outcomes for patients currently being treated. For example, the present study population showed a decrease in anti-Her2 targeted therapy use compared with the current treatment standards; however, as this factor applies for both BCS + RT and mastectomy, it is not expected to have biased the results. Meanwhile, the findings may be valid only in those with macrometastasis rather than micrometastasis in axilla, because only 3.8% of the patients received sentinel node biopsy. It has been shown that extensive pathologic assessment of sentinel nodes results in frequent identification of micrometastatic foci and the use of PMRT in patients with small-volume nodal disease should be conservative [28]. Third, the adjustment for registered characteristics might not exclude residual confounding, and selection bias still potentially had influence on the estimation of treatment effects.

## Conclusion

Based on our real world analyses, we found that postoperative radiotherapy rather than the surgery procedures was associated with superior survival outcomes in patients with T1–2N1M0 breast cancer. These findings need further validation.

## Supplementary Information

**Additional file 1: Table S1.** Baseline characteristics of patients in MRM without RT and BCS + RT groups before and after match. **Table S2.** Baseline characteristics of patients in MRM + RT and BCS + RT groups pre- and post-matched by propensity score analysis.

## Data Availability

The datasets used and/or analysed during the current study are available from the corresponding author on reasonable request.

## Abbreviations

BCS: Breast-conserving surgery
BCS + RT: Breast-conserving surgery plus radiotherapy
DFS: Disease-free-survival
ER: Estrogen receptor
HER2: Human epidermal growth factor receptor 2
LRR: Locoregional recurrence
MRM: Modified radical mastectomy
MRM + RT: Modified radical mastectomy plus radiotherapy
OS: Overall survival
RT: Radiotherapy
PMRT: Postmastectomy radiotherapy
PR: Progesterone receptor
PSM: Propensity score-matching
