# Supplementary material for: Radiotherapy plays an important role in improving the survival outcome in patients with T1–2N1M0 breast cancer – a joint analysis of 4262 real world cases from two institutions
Source: BMC Cancer. 2020 Nov 26;20:1155. doi: 10.1186/s12885-020-07646-y (PMC7691055; doi:10.1186/s12885-020-07646-y)
Supplement: Supplementary file 1 — Additional file 1: Table S1. Baseline characteristics of patients in MRM without RT and BCS + RT groups before and after match. Table S2. Baseline characteristics of patients in MRM + RT and BCS + RT groups pre- and post-matched by propensity score analysis. [file 12885_2020_7646_MOESM1_ESM.docx]

Table 1S. Baseline characteristics of patients in MRM without RT and BCS+RT groups before and after match.

|  | **No. (%)** | | | | ***P*** | | **No. (%)** | | | | ***P*** |
| --- | --- | --- | --- | --- | --- | --- | --- | --- | --- | --- | --- |
|  | **the entire cohort**  **(n=3430)** | **MRM without RT**  **(n=3026)** | **BCS+RT**  **(n=404)** |  | | **the matched cohort**  **(n=712)** | | | **MRM without RT**  **(n=356)** | **BCS+RT**  **(n=356)** |  |
| Year |  |  |  | < .001 | |  | |  | |  | .740 |
| 1999-2008 | 1637 (47.7) | 1532 (50.6) | 105 (26.0) |  | | 203 (28.5) | | 99 (27.8) | | 104 (29.2) |  |
| 2009-2014 | 1793 (51.5) | 1494 (49.4) | 299 (74.0) |  | | 509 (71.5) | | 257 (72.2) | | 252 (70.8) |  |
| Age (years) |  |  |  | < .001 | |  | |  | |  | .404 |
| ≤ 40 | 592 (17.3) | 468 (15.5) | 124 (30.7) |  | | 199 (27.9) | | 94 (26.4) | | 105 (29.5) |  |
| > 40 | 2838 (82.7) | 2558 (84.5) | 280 (69.3) |  | | 513 (72.1) | | 262 (73.6) | | 251 (70.5) |  |
| Tumor location |  |  |  | .730 | |  | |  | |  | .324 |
| Inner quadrant | 739 (21.5) | 649 (21.4) | 90 (22.3) |  | | 169 (23.7) | | 88 (24.7) | | 81 (22.8) |  |
| Other quadrants | 2644 (77.1) | 2337 (77.2) | 307 (76.0) |  | | 535 (75.1) | | 266 (74.7) | | 269 (75.6) |  |
| Unknown | 47 (1.4) | 40 (1.3) | 7 (1.7) |  | | 8 (1.1) | | 2 (0.6) | | 6 (1.7) |  |
| T stage |  |  |  | < .001 | |  | |  | |  | .419 |
| T1 | 1681 (49.0) | 1397 (46.2) | 284 (70.3) |  | | 489 (68.7) | | 250 (70.2) | | 239 (67.1) |  |
| T2 | 1749 (51.0) | 1629 (53.8) | 120 (29.7) |  | | 223 (31.3) | | 106 (29.8) | | 117 (32.9) |  |
| SLNB |  |  |  | < .001 | |  | |  | |  | .906 |
| No | 3285 (95.8) | 2968 (98.1) | 317 (78.5) |  | | 632 (88.8) | | 317 (89.0) | | 315 (88.5) |  |
| Yes | 145 (4.2) | 58 (1.9) | 87 (21.5) |  | | 80 (11.2) | | 39 (11.0) | | 41 (11.5) |  |
| No. of ALND |  |  |  | .964 | |  | |  | |  | .382 |
| ≤ 19 | 2170 (63.3) | 1914 (63.3) | 256 (63.4) |  | | 474 (66.6) | | 243 (68.3) | | 231 (64.9) |  |
| > 19 | 1260 (36.7) | 1112 (36.7) | 148 (36.6) |  | | 238 (33.4) | | 113 (31.7) | | 125 (35.1) |  |
| No. of positive nodes |  |  |  | 0.608 | |  | |  | |  | .569 |
| 1 | 1943 (56.6) | 1708 (56.4) | 235 (58.2) |  | | 420 (59.0) | | 208 (58.4) | | 212 (59.6) |  |
| 2 | 989 (28.8) | 881 (29.1) | 108 (26.7) |  | | 195 (27.4) | | 103 (28.9) | | 92 (25.8) |  |
| 3 | 498 (14.5) | 437 (14.4) | 61 (15.1) |  | | 97 (13.6) | | 45 (12.6) | | 52 (14.6) |  |
| Lymphovascular invasion |  |  |  | < .001 | |  | |  | |  | .369 |
| Yes | 381 (11.1) | 329 (10.9) | 52 (12.9) |  | | 83 (11.7) | | 36 (10.1) | | 47 (13.2) |  |
| No | 2810 (81.9) | 2462 (81.4) | 348 (86.1) |  | | 622 (87.4) | | 317 (89.0) | | 305 (85.7) |  |
| unknown | 239 (7.0) | 235 (7.8) | 4 (1.0) |  | | 7 (1.0) | | 3 (0.8) | | 4 (1.1) |  |
| Histological grade |  |  |  | < .001 | |  | |  | |  | .093 |
| I | 123 (3.6) | 94 (3.1) | 29 (7.2) |  | | 36 (5.1) | | 12 (3.4) | | 24 (6.7) |  |
| II | 1886 (55.0) | 1631 (53.9) | 255 (63.1) |  | | 462 (64.9) | | 244 (68.5) | | 218 (61.2) |  |
| III | 807 (23.5) | 714 (23.6) | 93 (23.0) |  | | 170 (23.9) | | 80 (22.5) | | 90 (25.3) |  |
| unknown | 614 (17.9) | 587 (19.4) | 27 (6.7) |  | | 44 (6.2) | | 20 (5.6) | | 24 (6.7) |  |
| Chemotherapy |  |  |  | < .001 | |  | |  | |  | .143 |
| No | 230 (6.7) | 227 (7.5) | 3 (0.7) |  | | 12 (1.7) | | 9 (2.5) | | 3 (0.8) |  |
| Yes | 3174 (92.5) | 2773 (91.6) | 401 (99.3) |  | | 700 (98.3) | | 347 (97.5) | | 353 (99.2) |  |
| unknown | 26 (0.8) | 26 (0.9) | 0 (0) |  | | 0 (0) | | 0 (0) | | 0 (0) |  |
| Chemotherapeutic drug |  |  |  | < .001 | |  | |  | |  | .454 |
| Taxane-based | 2106 (61.4) | 1766 (58.4) | 340 (84.2) |  | | 579 (81.3) | | 283 (79.5) | | 296 (83.1) |  |
| Others | 899 (26.2) | 851 (28.1) | 48 (11.9) |  | | 98 (13.8) | | 54 (15.2) | | 44 (12.4) |  |
| Unknown | 425 (12.4) | 409 (13.5) | 16 (4.0) |  | | 35 (4.89) | | 19 (5.3) | | 16 (4.5) |  |
| Hormone receptor & Hormonal therapy |  |  |  | < .001 | |  | |  | |  | .243 |
| negative & no | 682 (19.9) | 631 (20.9) | 51 (12.6) |  | | 101 (14.2) | | 52 (14.6) | | 49 (13.8) |  |
| positive & yes | 2340 (68.2) | 2015 (66.6) | 325 (80.4) |  | | 544 (76.4) | | 264 (74.2) | | 280 (78.7) |  |
| positive & no | 276 (8.0) | 259 (8.6) | 17 (4.2) |  | | 47 (6.6) | | 30 (8.4) | | 17 (4.8) |  |
| Unknown | 132 (3.8) | 121 (4.0) | 11 (2.7) |  | | 20 (2.8) | | 10 (2.8) | | 10 (2.8) |  |
| HER2 & Target therapy |  |  |  | < .001 | |  | |  | |  | .386 |
| negative & no | 2353 (68.6) | 2052 (67.8) | 301 (74.5) |  | | 523 (73.5) | | 259 (72.8) | | 264 (74.2) |  |
| positive & yes | 153 (4.5) | 118 (3.9) | 35 (8.7) |  | | 53 (7.4) | | 22 (6.2) | | 31 (8.7) |  |
| positive & no | 499 (14.5) | 462 (15.3) | 37 (9.2) |  | | 75 (10.5) | | 42 (11.8) | | 33 (9.3) |  |
| unknown | 425 (12.4) | 394 (13.0) | 31 (7.7) |  | | 61 (8.6) | | 33 (9.3) | | 28 (7.9) |  |

*Abbreviations:* MRM = modified radical mastectomy; BCS = breast-conserving surgery; RT = radiotherapy; SLNB = sentinel lymph node biopsy; ALND = axillary lymph node dissection; HER2 = human epidermal growth factor receptor 2

Table 2S. Baseline characteristics of patients in MRM + RT and BCS + RT groups pre- and post-matched by propensity score analysis.

|  | **No. (%)** | | | ***P*** | **No. (%)** | | | | ***P*** |
| --- | --- | --- | --- | --- | --- | --- | --- | --- | --- |
|  | **the entire cohort**  **(n=1236)** | **MRM+ RT**  **(n=832)** | **BCS+RT**  **(n=404)** |  | **the matched cohort**  **(n=488)** | | **MRM+RT**  **(n=244)** | **BCS+RT**  **(n=244)** |  |
| Year |  |  |  | < .001 |  |  | |  | 1.000 |
| 1999-2008 | 444 (35.9) | 339 (40.7) | 105 (26.0) |  | 140 (28.7) | 70 (28.7) | | 70 (28.7) |  |
| 2009-2014 | 792 (64.1) | 439 (59.3) | 299 (74.0) |  | 348 (71.3) | 174 (71.3) | | 174 (71.3) |  |
| Age (years) |  |  |  | .048 |  |  | |  | .843 |
| ≤ 40 | 334 (27.0) | 210 (25.2) | 124 (30.7) |  | 145 (29.7) | 74 (30.3) | | 71 (29.1) |  |
| > 40 | 909 (73.0) | 622 (74.8) | 280 (69.3) |  | 343 (70.3) | 170 (69.7) | | 173 (70.9) |  |
| Tumor location |  |  |  | .751 |  |  | |  | .930 |
| Inner quadrant | 260 (21.0) | 170 (20.4) | 90 (22.3) |  | 110 (22.5) | 55 (22.5) | | 55 (22.5) |  |
| Other quadrants | 955 (77.3) | 648 (77.9) | 307 (76.0) |  | 371 (76.0) | 185 (75.8) | | 186 (76.2) |  |
| Unknown | 21 (1.7) | 14 (1.7) | 7 (1.7) |  | 7 (1.4) | 4 (1.6) | | 3 (1.2) |  |
| T stage |  |  |  | <0.001 |  |  | |  | .780 |
| T1 | 612 (49.5) | 328 (39.4) | 284 (70.3) |  | 302 (61.9) | 153 (62.7) | | 149 (61.1) |  |
| T2 | 624 (50.5) | 504 (60.6) | 120 (29.7) |  | 186 (38.1) | 91 (37.3) | | 95 (38.9) |  |
| SLNB |  |  |  | < .001 |  |  | |  | .828 |
| No | 1131 (91.5) | 814 (97.8) | 317 (78.5) |  | 466 (95.5) | 232 (95.1) | | 234 (95.9) |  |
| Yes | 105 (8.5) | 18 (2.2) | 87 (21.5) |  | 22 (4.5) | 12 (4.9) | | 10 (4.1) |  |
| No. of ALND |  |  |  | .340 |  |  | |  | .921 |
| ≤ 19 | 807 (65.3) | 551 (66.2) | 256 (63.4) |  | 345 (70.7) | 172 (70.5) | | 173 (70.9) |  |
| > 19 | 429 (34.7) | 281 (33.8) | 148 (36.6) |  | 143 (29.3) | 72 (29.5) | | 71 (29.1) |  |
| No. of positive nodes |  |  |  | < .001 |  |  | |  | .964 |
| 1 | 490 (39.6) | 255 (30.6) | 235 (58.2) |  | 230 (47.1) | 115 (47.1) | | 115 (47.1) |  |
| 2 | 383 (31.0) | 275 (33.1) | 108 (26.7) |  | 158 (32.4) | 78 (32.0) | | 80 (32.8) |  |
| 3 | 363 (29.4) | 302 (36.3) | 61 (15.1) |  | 100 (20.5) | 51 (20.9) | | 49 (20.1) |  |
| Lymphovascular invasion |  |  |  | < .001 |  |  | |  | .185 |
| Yes | 193 (15.6) | 141 (16.9) | 52 (12.9) |  | 79 (16.2) | 45 (18.4) | | 34 (13.9) |  |
| No | 995 (80.5) | 647 (77.8) | 348 (86.1) |  | 399 (81.8) | 192 (78.7) | | 207 (84.8) |  |
| unknown | 48 (3.9) | 44 (5.3) | 4 (1.0) |  | 10 (2.0) | 7 (2.9) | | 3 (1.2) |  |
| Histological grade |  |  |  | < .001 |  |  | |  | .491 |
| I | 39 (3.2) | 10 (1.2) | 29 (7.2) |  | 8 (1.6) | 2 (0.8) | | 6 (2.5) |  |
| II | 659 (53.3) | 404 (48.6) | 255 (63.1) |  | 304 (62.3) | 152 (62.3) | | 152 (62.3) |  |
| III | 316 (25.6) | 223 (26.8) | 93 (23.0) |  | 131 (26.8) | 65 (26.6) | | 66 (27.0) |  |
| unknown | 222 (18.0) | 195 (23.4) | 27 (6.7) |  | 45 (9.2) | 25 (10.2) | | 20 (8.2) |  |
| Chemotherapy |  |  |  | .786 |  |  | |  | .499 |
| No | 1222 (98.9) | 9 (1.1) | 3 (0.7) |  | 2 (0.4) | 0 (0) | | 2 (0.8) |  |
| Yes | 12 (1.0) | 821 (98.7) | 401 (99.3) |  | 486 (99.6) | 244 (100) | | 242 (99.2) |  |
| unknown | 2 (0.2) | 2 (0.2) | 0 (0) |  | NA | NA | | NA |  |
| Chemotherapeutic drug |  |  |  | < .001 |  |  | |  | .123 |
| Taxane-based | 918 (74.3) | 578 (69.5) | 340 (84.2) |  | 414 (84.8) | 199 (81.6) | | 215 (88.1) |  |
| Others | 254 (20.6) | 206 (24.8) | 48 (11.9) |  | 63 (12.9) | 39 (16.0) | | 24 (9.8) |  |
| Unknown | 64 (5.2) | 48 (5.8) | 16 (4.0) |  | 11 (2.3) | 6 (2.5) | | 5 (2.0) |  |
| Hormone receptor & Hormonal therapy |  |  |  | < .001 |  |  | |  | .458 |
| negative & no | 282 (22.8) | 231 (27.8) | 51 (12.6) |  | 90 (18.4) | 49 (20.1) | | 41 (16.8) |  |
| positive & yes | 847 (68.5) | 522 (62.7) | 325 (80.4) |  | 369 (75.6) | 180 (73.8) | | 189 (77.5) |  |
| positive & no | 53 (4.3) | 36 (4.3) | 17 (4.2) |  | 13 (2.7) | 5 (2.0) | | 8 (3.3) |  |
| Unknown | 54 (4.4) | 43 (5.2) | 11 (2.7) |  | 16 (3.3) | 10 (4.1) | | 6 (2.5) |  |
| HER2 & Target therapy |  |  |  | < .001 |  |  | |  | .987 |
| negative & no | 794 (64.2) | 493 (59.3) | 301 (74.5) |  | 366 (75.0) | 182 (74.6) | | 184 (75.4) |  |
| positive & yes | 115 (9.3) | 80 (9.6) | 35 (8.7) |  | 39 (8.0) | 19 (7.8) | | 20 (7.2) |  |
| positive & no | 157 (12.7) | 120 (14.4) | 37 (9.2) |  | 45 (9.2) | 23 (9.4) | | 20 (9.0) |  |
| unknown | 170 (13.8) | 139 (16.7) | 31 (7.7) |  | 38 (7.8) | 20 (8.2) | | 18 (7.4) |  |

*Abbreviations:* MRM = modified radical mastectomy; BCS = breast-conserving surgery; RT = radiotherapy; SLNB = sentinel lymph node biopsy; ALND = axillary lymph node dissection; HER2 = human epidermal growth factor receptor 2
